# A Treatise on the Science and Practice of Midwifery

**Published:** 1877-03

**Authors:** 


### BIBLIOGRAPHICAL.

A Treatise on the Science and Practice of Midwifery. By W. S. Playfair, M.D., F.R.C.D., Professor of Obstetric Medicine in Kings College Hospital; Examiner in Midwifery to the University of London; late Examiner in Midwifery to the Royal College of Physicians; and Vice-President of the Obstetrical Society of London. Philadelphia: Henry C. Lea. 1876.

"The author's object has been to place in the hands of his readers an epitome of the science and practice of midwifery, which embodies all recent advances." The work is divided into five parts, viz: I. Anatomy and Physiology of the organs concerned in Parturition. II. Pregnancy. III. Labor. IV. Obstetric operations. V. The Puerperal State.

The perusal of this work has afforded us great pleasure as well as profit, since it is so fully up to the most advanced ideas in obstetrics, and in several important particulars, making decided departures from the traditionary paths. It indeed takes rank with Leishman and Schrœder, and leaves in the back ground the valued old land marks, Ramsbotham, Cazeaux, etc.

The chapter on obstetric operations is especially interesting and valuable. We are taught to discard the long established teachings in the application of the forceps to the sides of the child's head, and to apply them always to the sides of the pelvis. In this, the author follows Robert Barnes, in Great Britan and the German obstetricians in general, who teach that the position of the head should be practically disregarded and the forceps applied in relation to the pelvis. Could the author have overcome the prejudices of habit and education sufficiently to have adopted also the American obstetric position "on the back" instead of that "on the side," for forceps operations, he would have overcome another common and objectionable feature. The chapter on "anæsthesia in labor" is especially admirable. "There is one cardinal rule to be remembered in giving chloroform during the propulsive stage, and that is, that it should be administered intermittingly, and never continuously, When the pain comes on a few drops may be scattered over a Skinner's inhaler, which affords one of the best means of administering it in labor, or placed within the folds of a handkerchief twisted into the form of a cone. During the acme of the pain the patient inhales it freely, and at once experiences a sense of great relief, and as soon as the pain dies away the inhaler should be removed. In the interval between the pains the effect of the drug passes off, so that the higher degrees of anæsthesia should never be produced. * * * This intermittent administration constitutes the peculiar safety of chloroform administration in labor, and it is a fortunate circumstance that as yet there is, I believe, no case on record of death during the inhalation of chloroform for obstetric purposes." In the early stage of labor chloral is recommended in preference to chloroform as interfering less with the efficacy of the pains and as possessing superior properties in the dilatation of the cervix.

In reference to the much confused and disputed question of puerperal fever, the author, we are gratified to see, has decided opinions, and takes bold and positive ground against the dogma of "an essential zymotic disease, *sui generis*, which affects lying-in-women only." The term "puerperal fever" is discarded, and in its stead the more appropriate one of "puerperal septicæmia" adopted. We are told that "the whole tendency of recent investigation is daily rendering it more and more certain that obstetricians have been led into error by the special virulence and intensity of the disease, and that they have erroneously considered it to be something special to the puerperal state, instead of recognizing in it a form of septic disease practically identical with that which is familiar with surgeons under the name of pyæmia or septicæmia"

We take pleasure in recommending the work to the student and the practitioner. V. H. T.

